# Circular Polymerase Extension Cloning of Complex Gene Libraries and Pathways

**DOI:** 10.1371/journal.pone.0006441

**Published:** 2009-07-30

**Authors:** Jiayuan Quan, Jingdong Tian

**Affiliations:** Department of Biomedical Engineering & Institute for Genome Sciences and Policy, Duke University, Durham, North Carolina, United States of America; Instituto Butantan, Brazil

## Abstract

High-throughput genomics and the emerging field of synthetic biology demand ever more convenient, economical, and efficient technologies to assemble and clone genes, gene libraries and synthetic pathways. Here, we describe the development of a novel and extremely simple cloning method, circular polymerase extension cloning (CPEC). This method uses a single polymerase to assemble and clone multiple inserts with any vector in a one-step reaction *in vitro*. No restriction digestion, ligation, or single-stranded homologous recombination is required. In this study, we elucidate the CPEC reaction mechanism and demonstrate its usage in demanding synthetic biology applications such as one-step assembly and cloning of complex combinatorial libraries and multi-component pathways.

## Introduction

Molecular cloning is a foundational technology for molecular biology and biotechnology. Pioneered by the restriction digestion and ligation based method [1]–[3], new cloning technologies have continuously been invented and evolved to suit various requirements and applications. Depending on whether specific sites or sequences are used in the insert and the vector for cloning, cloning methods can be broadly divided into two categories: sequence-dependent and sequence-independent. Sequence-dependent cloning is based either on restriction digestion and ligation, or site-specific recombination, such as the Univector plasmid-fusion system [4] and Gateway [5], [6]. Sequence-independent cloning is largely based on homologous recombination and includes methods such as ligase-free [7] or ligation-independent cloning (LIC) [8], LIC with Uracil DNA glycosylase (UDG or USER cloning) [9], [10], MAGIC [11], SLIC [12], In-Fusion (Clontech) [13], and PIPE [14]. Although these methods all have their own special characteristics and advantages, new developments especially the emergence of synthetic biology have put ever increasing demand for more accurate, efficient, convenient and economical cloning technologies for purposes such as creating complex combinatorial synthetic gene libraries, gene circuits and metabolic pathways.

For synthetic biology applications involving high-complexity or multi-fragment cloning, sequence-dependent methods are generally inconvenient because they require unique and specific sites in both the insert and the vector in order to generate the initial plasmids [4]–[6]. For this reason, the more flexible sequence-independent cloning methods are preferred. However, such methods usually require generating complementary single-stranded overhangs in both the insert and vector fragments, with or without RecA-mediation [8], [12], [14]. And some of these methods are not strictly sequence-independent because they require the presence or absence of specific nucleotides at certain positions in the overlapping region [8], [15]. The generation of complementary single-stranded overhangs takes additional preparation steps and often uses expensive enzyme systems. These manipulations generally require large amounts of DNA at the beginning and tend to have insufficient efficiency for library cloning. Furthermore, the annealing step in these methods is normally performed at ambient temperature, which allows non-specific hybridization among single-stranded overhangs and lead to frequent assembly errors in multi-fragment cloning. Therefore, for the demanding tasks of assembling and cloning complex synthetic gene libraries and pathways, further improvements on accuracy and efficiency over existing methods would be highly desirable. For routine and high-throughput cloning, fewer steps and lower cost is always a significant improvement.

Polymerase extension is the basis of the polymerase chain reaction (PCR) used for amplification of DNA sequences. The same principle is also used for gene assembly with overlapping oligonucleotides or gene fragments [16]–[18]. However, to our knowledge, there has been no reported gene cloning method which solely relies on this mechanism. Here we report the development of a much simplified sequence-independent cloning technology based entirely on the polymerase extension mechanism. This method extends overlapping regions between the insert and vector fragments to form a complete circular plasmid and is therefore named “Circular Polymerase Extension Cloning”. In the current study, we elucidate the reaction mechanism and demonstrate the broad utility and advantages of CPEC in cloning of synthetic genes, complex combinatorial libraries and metabolic pathways. We performed extensive tests on CPEC and recommend it as one of the most convenient, economical, and accurate cloning method.

## Results

### Single gene cloning using CPEC

**Figure 1:**
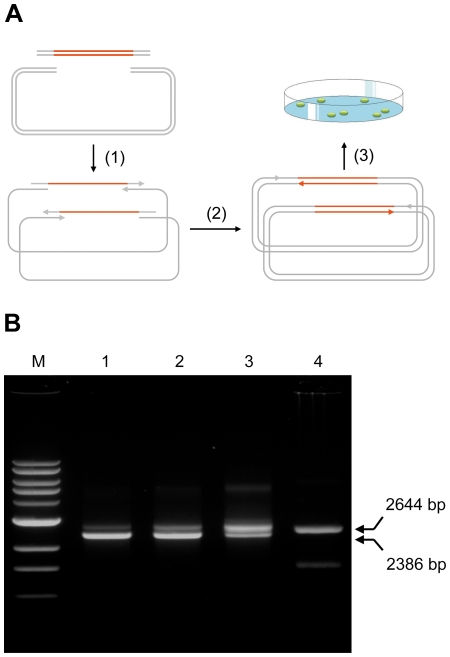
Gene cloning using CPEC. (A) A schematic diagram of the proposed CPEC mechanism for cloning an individual gene. The vector and the insert share overlapping regions at the ends. After denaturation and annealing (Step 1), the hybridized insert and vector extend using each other as a template until they complete a full circle and reach their own 5′-ends (Step 2). The final completely assembled plasmid has two nicks, one on each strand, at the positions marked by an arrow head. They can be used for transformation (Step 3) with or without further purification. For library cloning, the cycle maybe repeated in order to increase the yield of complete plasmids. (B) CPEC cloning of the *lacZα* gene. The image shows gel electrophoresis analysis of the CPEC reaction product after 1, 2 and 5 cycles (lanes 1–3). 5 µl of the reaction was separated on a 0.8% agarose gel and visualized after ethidium bromide staining. The assembled full-length plasmid was 2644 bp; the empty vector, 2386 bp. A sequence verified, full-length plasmid purified from a bacteria colony was used as a positive control (lane 4). The upper band (2644 bp) represented the relaxed circular form and the fast-migrating lower band, the closed circular form of the plasmid. The molecular weight marker used in this figure was NEB 1 kb DNA ladder (lane M).

Existing sequence-independent cloning methods require generating complementary single-stranded overhangs between the insert and the vector, a time-consuming and expensive process. We reasoned that it might be possible to eliminate this requirement by using the polymerase extension mechanism to extend double-stranded overlapping insert and vector to form a complete plasmid (Fig. 1A). In this mechanism, the insert and the vector share overlapping sequences on both ends. After denaturation and annealing, the insert and the vector will hybridize and extend using each other as a template to form a complete double-stranded plasmid, leaving only one nick in each strand.

To confirm the validity of this mechanism, we first attempted cloning of a simple test gene, *lacZα*, into a modified pUC19 expression vector (see Methods S1 for sequence information). The vector was linearized using either restriction digestion or PCR method. We added sequences on both ends of the *lacZα* gene to overlap with the ends of the linearized vector. The overlapping regions between the inset and the vector were designed to have similar melting temperatures (Tm), which were typically between 60–70°C (see Methods S1 for sequence information). We mixed the linearized vector with the *lacZα* gene without adding any PCR primers in an otherwise typical PCR reaction mixture.

We performed CPEC cloning as we would perform one cycle of PCR using a high-fidelity DNA polymerase. The reaction involved a brief denaturation step to denature the double-stranded insert and the linear vector, an annealing step for the overlapping ends of the insert and the vector to hybridize, and an extension step to form a complete plasmid (see Methods). We examined a small aliquot of the reaction mixture using DNA agarose gel electrophoresis and used another small amount for transformation.

The gel electrophoresis results showed formation of a significant amount of vector-insert merging product (Fig. 1B, lane 1, upper band) after only one reaction cycle with equal molar concentrations of the vector and the insert. The amount of this product increased proportionally after 2 and 5 reaction cycles (Fig. 1B, lanes 2 and 3, upper band). This band appeared to migrate at the same position as the purified full-length plasmid in its nicked relaxed conformation (Fig. 1B, lane 4, upper band). An examination of the transform results found that approximately 100% of the colonies showed blue color, indicating minimal cloning error or carry-over of intact vectors. Sequencing results of randomly picked colonies confirmed that the cloning reaction happened exactly as expected, with no mutations at the cloning junctions.

### Gene library cloning using CPEC

For individual gene cloning, we determined that one cycle of CPEC reaction was optimal. For complex gene library cloning where sufficient numbers of clones need to be obtained in order to maintain the complexity of the library, more cycles of CPEC reaction might be needed. To determine the optimal library cloning conditions with CPEC, we examined the cloning a synthetic library containing codon variants of the *lacZα* gene, which was designed and synthesized for studying the effects of synonymous codon usage on protein expression. We selected the *lacZα* gene because we could use the blue or white color of the colonies to demonstrate the cloning and expression results. For this complex gene library, no convenient restriction sites could be found for cloning into the modified pUC19 expression vector without cutting a fraction of the insert sequences. Therefore, a sequence-independent cloning method must be used.

**Figure 2:**
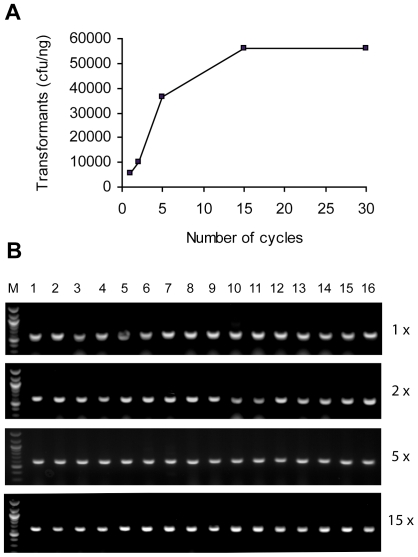
Gene library cloning using CPEC. (A) Cloning efficiency of CPEC at different cycles using the *lacZα* codon variants library. (B) Examination of the length of the insert from 64 independent colonies by single-colony PCR. The colonies came from cells transformed with CPEC reaction products after 1, 2, 5, and 15 cycles. The vector-insert ratio in the CPEC reactions was 1∶1. The length of the amplicon with one insert was 592 bp. Single-copy inserts were found in all of the 64 colonies examined. The molecular weight marker used in this figure was NEB 100 bp DNA ladder (lane M).

We performed CPEC cloning of the library and determined the cloning efficiency at different cycle numbers (see Methods). The results indicated that 5,333 transformants were obtained from one nanogram of insert after only one cycle, which was sufficient for routine library cloning. The number of transformants obtained peaked at around 15 cycles and reached 56,000 colony forming units (c.f.u.) per nanogram of insert (Fig. 2A). As a comparison, we typically achieved approximately 1,200∼1,500 c.f.u per nanogram of control insert using the ligation method. Approximately 100% of the colonies on the positive control plate transformed with *wt*-*lacZα* gene showed blue color, while the colonies with codon variations demonstrated, as expected, a wide range of blue color intensities. We isolated and sequenced plasmids from several hundred independent colonies which showed different intensities of the blue color. The sequencing results showed the presence of a distinct codon variant sequence of *lacZα* in every plasmid, which demonstrated correct and unbiased cloning of the complex library using CPEC. We further investigated the percentage of clones that might carry more than one insert after different number of CPEC cycles by single-colony PCR using primers on the vector (Fig. 2B). Sixteen colonies were randomly picked from plates culturing cells transformed with CPEC reaction product after 1, 2, 5 and 15 cycles. It was found that all 64 plasmids examined contained the correct, single-copy insert. This result suggested that the CPEC cloning mechanism was highly specific and did not favor carry-over of concatemers, if any, into the final clones.

### Combinatorial library cloning using CPEC

**Figure 3:**
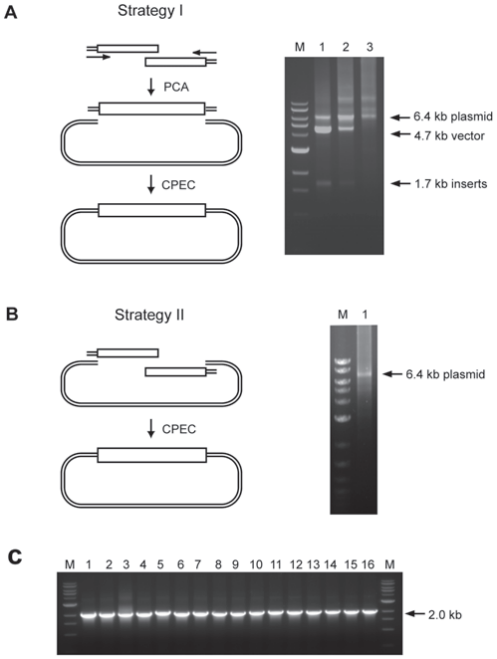
Combinatorial gene library cloning using CPEC. (A) A multi-step strategy combining PCA and CPEC for constructing combinatorial synthetic gene library. As shown in the schematic diagram on the left, in the first step, polymerase cycle assembly (PCA) is used to assemble two sub-libraries into a full-length library; in the second step, CPEC is employed to clone the full-length *gp120* gene library inserts (1.7 kb) into the vector (4.7 kb). The gel electrophoresis picture on the right shows the analysis of CPEC progression after 5, 10 and 20 cycles (lanes 1–3). The molecular weight marker used in this figure was NEB 1 kb DNA ladder (lane M). (B) A one-step strategy of constructing combinatorial library using CPEC. The two sub-libraries and the linear vector were mixed in equal concentrations in the CPEC reaction. After 25 cycles, the reaction product was analyzed by 0.8% agarose gel electrophoresis. Arrow marked the 6.4-kb band representing the full-length plasmid. The molecular weight marker used in this figure was NEB 2-log DNA ladder. (C) Gel electrophoresis analysis of inserts amplified from 16 independent colonies from the *gp120* library cloned using the one-step CPEC strategy. The molecular weight marker used in this figure was NEB 1 kb DNA ladder.

Construction of combinatorial library is extremely useful for synthetic biology and molecular evolution. We designed and tested two strategies of constructing complex combinatorial synthetic gene libraries using CPEC. The first strategy was to assemble the full-length inserts from shorter fragments first, followed by cloning the pre-assembled full-length inserts into a vector by CPEC. The second strategy was to combine the assembly and cloning steps into one CPEC reaction (Fig. 3).

For these tests, we selected a synthetic library which contained codon variants of the HIV envelope gene, *gp120*. The 1.7-kb full-length codon variant library was divided into two fragments of approximately equal lengths, which were synthesized separately (see Methods S1 for sequence information). The two fragments and the vector were designed to share overlapping sequences with similar melting temperatures. To test the first strategy, we preassembled the 1.7-kb combinatorial library using a two-step polymerase cycling assembly (PCA) reaction [19] (see Methods) and then mixed the insert with the linearized vector and performed a multi-cycle CPEC reaction. An aliquot of the reaction was taken after 5, 10 and 20 cycles, respectively, and the reaction products were analyzed by gel electrophoresis (Fig. 3A, lanes 1–3). The results indicated that after 5 cycles of CPEC, a significant amount of the full-length 6.4-kb plasmid had formed. By 10 cycles, approximately 80% of the 1.7-kb inserts had merged with the vector. After 20 cycles, all free inserts and vector DNA had merged to form the complete plasmid.

Next, we tested one-step combinatorial assembly and cloning of the library from two sub-libraries using CPEC. We mixed the two insert libraries with the linearized vector in equal molar concentrations and performed 25 cycles of CPEC. The annealing step was carefully controlled in terms of annealing temperature and cooling rate in order to achieve highest hybridization efficiency and accuracy. A single band representing the 6.4-kb complete plasmid was clearly visible in gel electrophoresis analysis (Fig. 3B). We then transformed an aliquot of the reaction mixture directly into competent cells. Approximately 2.43×10^5^ colonies were obtained from one picomole of vector DNA. We randomly picked independent colonies on the plate and performed single-colony PCR to verity the presence of the correct insert. The result showed that all 16 colonies examined contained the full-length insert, indicating a 100% cloning efficiency (Fig. 3C).

### Multi-component pathway assembly using CPEC

**Figure 4:**
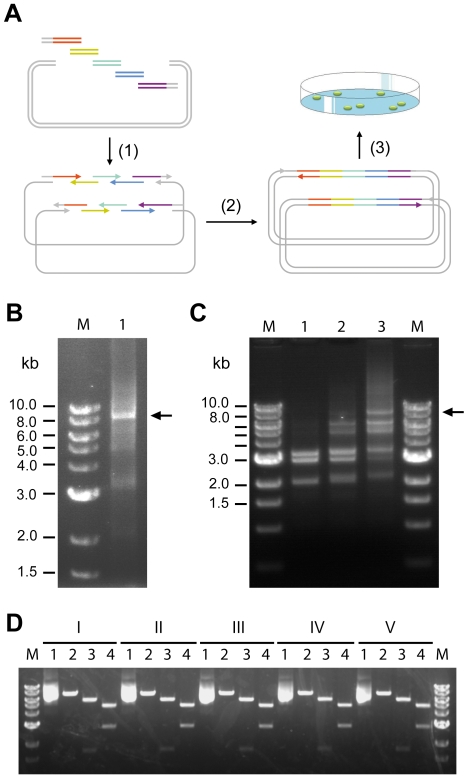
Assembly of multi-component pathway using CPEC. (A) A schematic diagram of the multi-way CPEC. Any two neighboring fragments share an overlapping region with identical Tm. Multiple cycles are usually needed to drive the reaction to completion. The positions of the two nicks (arrow head) in the final completely assembled plasmid may vary depending on the number, lengths, and sequences of the fragments. (B) Gel electrophoresis analysis of the final assembly product after a 20-cycle CPEC. 5 µl of the reaction was separated on a 0.8% agarose gel and visualized after ethidium bromide staining. The full-length plasmid was 8360 bp. (C) Gel electrophoresis analysis of the multi-way CPEC reaction. 5 µl was taken out of the reaction after 2, 5, and 10 cycles and separated on a 0.8% agarose gel (lanes 1, 2 and 3). The starting lengths of the four fragments were 3280, 2959, 2040, and 171 bp, respectively. The 171-bp band was not visible. (D) Restriction mapping of the isolated plasmids derived from the CPEC reaction. Plasmid DNA from five independent colonies (I-V) were digested with BamHI (lane 2, 8.4 kb), BamHI-XhoI (lane 3, 6.6 kb and 1.8 kb), and NdeI (lane 4, 5.4 kb and 3 kb). Purified plasmids not subjected to restriction digestion are shown in lane 1. The molecular weight marker used in this figure was NEB 1 kb DNA ladder.

We then tested if CPEC can be applied for efficient assembly and cloning of multi-component pathways in a single reaction. The proposed multi-way cloning mechanism is shown in Figure 4A. Unlike single-insert CPEC, which only require one cycle, multi-way CPEC usually requires multiple cycles to assemble a full-length product. However, unlike PCR, multi-cycle CPEC is not an amplification process, therefore will not accumulate or propagate errors generated by the DNA polymerase.

We tested multi-way CPEC by constructing and cloning a metabolic pathway for synthesizing a biodegradable plastic material, poly(3HB-co-4HB) in *E. coli*. The pathway consisted of four genes and additional regulatory elements. To construct the plasmid, we needed to assemble four PCR fragments of various lengths: 3280, 2959, 2047, and 171 bp. The total length of the ensemble was 8360 bp (see Methods S1 online for sequence information). We mixed these fragments in equal molar concentrations and carried out 20 cycles of CPEC reaction. Gel electrophoresis analysis showed the formation of a single prominent band of approximately 8.4 kb, representing the full-length plasmid (Fig. 4B). To dissect the formation process of the full-length plasmid in a multi-cycle CPEC reaction, we analyzed the reaction intermediates after 2, 5, and 10 cycles (Fig. 4C, lanes 1–3). Discrete bands representing extension products joining neighboring pieces to form longer and longer intermediates were clearly visible. The 8.4-kb full-length band was already strong by 10 cycles as the lengths of the intermediate bands shifted upward. The results supported the proposed mechanism (Fig. 4A) and suggested that multiple cycles are necessary in order to drive the reaction into completion.

To assess the quality of the cloned plasmid, we transformed 1.25 µl of the 20-cycle reaction mixture (∼20 ng of DNA) into competent *E. coli* cells and plated aliquots of the cells on chloramphenicol plates containing Nile Red. We calculated that approximately 1,000 transformants were obtained from 0.1 µl of the 20-cycle reaction. 100% of the colonies turned pink color, suggesting the presence of a functional pathway [20], [21]. We isolated plasmids from randomly picked colonies and performed restriction mapping. The results confirmed that all colonies examined contained the full-length plasmids (8.4 kb) with individual components at their expected positions (Fig. 4D).

## Discussion

Unlike any other cloning method, CPEC relies solely on the simple and robust polymerase extension mechanism to clone individual genes, libraries, or multiple fragments. In a single closed-tube reaction, the insert and vector fragments are first heat denatured, then annealed at elevated temperatures to ensure specific hybridization of overlapping regions, and finally extended to form complete plasmids, leaving only one nick in each strand. The fully-formed relaxed double-stranded plasmids are then efficiently introduced into *E. coli* cells where the nicks are sealed and covalently closed plasmids are formed. The most significant advantages of CPEC include accuracy, efficiency, convenience and cost-effectiveness in complex library and pathway assembly.

For routine single-gene cloning, one denature-annealing-extension cycle is sufficient and optimal, which can be completed in five minutes. For library cloning where sufficient numbers of clones need to be obtained in order to maintain the complexity of the library, CPEC offers the unique advantage of being able to perform multiple cycles to maximize the total number of clones constructed without using excessive amounts of vector DNA. We recommend 2–5 cycles depending on library complexity. For multi-fragment cloning, 5–25 cycles maybe used depending on the number of fragments.

Unlike PCR, CPEC is not an amplification process and therefore will not accumulate mutations. However, excessive numbers of cycles should be avoided in order to minimize possible concatemer formation. In cases where concatemers may form, the cloning efficiency will not be significantly affected because concatemers usually do not have the correct complementary ends for efficient circularization and therefore will not form covalently closed plasmids in the cells.

Only PCR polymerases with no strand displacement activity should be used in CPEC reactions to avoid long concatermer formation or other cloning artifacts. Many of the commercially available PCR polymerases belong to this category. The Phusion DNA polymerase was used in this study due to its robustness, speed and accuracy. The reaction conditions may need to be adjusted if other polymerases are used, especially the extension time. PCR polymerases with low efficiency or low fidelity should be avoided for demanding cloning tasks using CPEC.

Compared to sequence-dependent cloning methods, such as those mediated by restriction-ligation or site-specific recombination, CPEC has the advantage of complete flexibility with respect to sequence junctions. Compared to other sequence-independent cloning methods, in addition to enjoying all of their benefits, CPEC offers other significant advantages. First, CPEC eliminates the extra steps or enzymes required by other sequence-independent cloning methods to generate single-stranded regions for annealing. For example, in LIC, overlapping sequences lacking a particular dNTP are added to the insert by PCR and complementary 12-nt single-stranded regions in both the insert and the vector are generated by T4 DNA polymerase treatment in the presence of that particular dNTP. In UDG-based methods, a ribonucleotide U replaces a T in the PCR primers used to add overlapping sequences to the insert and subsequent treatment with UDG enzyme generates single-stranded ends in both the insert and the vector for annealing. In SLIC, T4 DNA polymerase treatment or incomplete PCR with two pairs of primers are used to generate mixed products containing ss-overlapping regions. In PIPE, a different version of incomplete PCR is used so that some PCR products are not fully extended and therefore leave heterogeneous single-stranded regions toward the ends. These extra preparation steps take more time, more DNA, and many require extra expensive enzymes or reagents. In contrast, CPEC uses double-stranded overlapping inserts and vector directly without any treatment. The whole single-cycle CPEC reaction can be completed in 5 minutes and uses only a PCR polymerase, making CPEC one of the most convenient, economical and versatile cloning methods, which can also be easily adapted for high-throughput cloning.

Another notable advantage of CPEC is its high cloning accuracy and efficiency, which makes it uniquely suitable for complex, combinatorial, multi-fragment or multi-library cloning. In CPEC, all overlapping regions among fragments are designed to have similar high melting temperatures (typically 55–70°C) so annealing between fragments can be very specific. This is most desirable for complex, combinatorial or multi-fragment cloning where non-specific annealing can cause cloning errors. It is our experience that typically 95–100% of CPEC-generated colonies contain the correct inserts, including multi-way assembly. All other sequence-independent cloning methods use ambient annealing temperatures and, as a result, the specificity and success rate of multi-way cloning can be significantly compromised.

The high cloning efficiency of CPEC, especially for multi-way or complex library cloning, comes from a combination of two special features. First, CPEC forms covalently joined complete plasmids *in vitro*. Secondly, multiple CPEC cycles can drive the reaction into near completion. In contrast, all other sequence-independent cloning methods either loosely anneal fragments without covalent bonding or allow only a small fraction of the fragments to form plasmids due to the low efficiency of multi-fragment hybridization.

With increasing demands for complex or combinatorial library cloning and multi-fragment gene pathway and network assembly, we expect CPEC to play a significant role in various applications of synthetic biology. It will enable rapid and high-throughput construction of combinatorial libraries, gene circuits and pathways. It will also liberate researchers from tedious and time-consuming everyday cloning tasks.

## Materials and Methods

### CPEC

We obtained linear vectors with PCR amplification and gel purified it using E.Z.N.A gel extraction kits (Omega Bio-Tek). We added vector-overlapping sequences onto the *lacZα* gene (with a C-terminal His6 tag) using PCR (see Methods S1 for primer sequences) and gel purified the insert. 200 ng of the linear vector was mixed with insert DNA at equal molar ratio in a 20 µl volume containing Phusion DNA polymerase reaction mixture (Finnzymes). We denatured the insert and vector mixture at 98°C for 30 seconds, annealed them at 55°C for 30 seconds, and performed polymerase extension for 15 seconds per kb according to the length of the longest piece. We normally added an extra extension period equivalent to 1–2 times of the required extension step in the end. For average-sized vectors and inserts, the total reaction time was less than 5 minutes. We transformed 1–4 µl of the mixture into 50 µl of chemically competent GC5α cells and plated a fraction of them on carbenicillin plates with 2% X-gal.

### Multi-cycle CPEC for library cloning

We set up the cloning reaction exactly the same way as in single-cycle CPEC. After the initial 30 second denaturation step, we performed multiple cycles each consisted of 10 seconds denaturation at 98°C, 30 seconds annealing at 55°C, and extension at 72°C for 20–30 seconds per kb according to the length of the longest piece. We ended the reaction with an extra 5 minutes of extension. We transformed a fraction of the reaction mixture into cells and plated an aliquot of the cells on a carbenicillin plate with 2% X-gal.

### Combinatorial library cloning

For the strategy combining PCA with CPEC, we set up the PCA reaction by mixing 100 ng of each sub-library (VacF1 and VacF2) with the Phusion reaction mixture in a 50 µl volume. After an initial 30 seconds denaturation at 98°C, we performed 5 cycles of PCA which consisted of 7 seconds denaturation at 98°C, 30 seconds annealing at 52°C, and extension at 72°C for 20 seconds, and completed with an extension step at 72°C for 5 minutes. We used 0.5 µl of the PCA reaction as a template and performed 30 cycles of PCR amplification of the full-length library using GP140R and GP140L primers (Methods S1) and the Phusion enzyme. We performed multi-cycle CPEC using identical conditions as described except that during the annealing step, we applied slow ramping at 0.1°C/second from 70°C to 55°C before annealing at 55°C for 30 seconds.

### Multi-way CPEC

We mixed equal molar concentrations of the insert and vector fragments for multi-way CPEC. We used extension time which was sufficient to cover the full-length of the plasmid. Otherwise the reaction condition was identical to the multi-cycle CPEC. We transformed 1.25 µl of the reaction into 50 µl chemically competent DH5α cells and plated 100–200 µl aliquots from 1 ml culture on 2% agar plates containing 20 µg/ml chloramphenicol and 0.5 µg/ml Nile Red.

### Additional Methods

Information about plasmid construction, vector, inserts and primer sequences is available in Supplementary Methods S1.

## Supporting Information

Methods S1 (0.09 MB DOC)

## References

[1] Smith HO, Wilcox KW (1970). A restriction enzyme from Hemophilus influenzae. I. Purification and general properties.. J Mol Biol.

[2] Danna K, Nathans D (1971). Specific cleavage of simian virus 40 DNA by restriction endonuclease of Hemophilus influenzae.. Proc Natl Acad Sci U S A.

[3] Cohen SN, Chang AC, Boyer HW, Helling RB (1973). Construction of biologically functional bacterial plasmids in vitro.. Proc Natl Acad Sci U S A.

[4] Liu Q, Li MZ, Leibham D, Cortez D, Elledge SJ (1998). The univector plasmid-fusion system, a method for rapid construction of recombinant DNA without restriction enzymes.. Curr Biol.

[5] Hartley JL, Temple GF, Brasch MA (2000). DNA cloning using in vitro site-specific recombination.. Genome Res.

[6] Walhout AJ, Temple GF, Brasch MA, Hartley JL, Lorson MA (2000). GATEWAY recombinational cloning: application to the cloning of large numbers of open reading frames or ORFeomes.. Methods Enzymol.

[7] Shuldiner AR, Scott LA, Roth J (1990). PCR-induced (ligase-free) subcloning: a rapid reliable method to subclone polymerase chain reaction (PCR) products.. Nucleic Acids Res.

[8] Aslanidis C, de Jong PJ (1990). Ligation-independent cloning of PCR products (LIC-PCR).. Nucleic Acids Res.

[9] Rashtchian A (1995). Novel methods for cloning and engineering genes using the polymerase chain reaction.. Curr Opin Biotechnol.

[10] Bitinaite J, Rubino M, Varma KH, Schildkraut I, Vaisvila R (2007). USER friendly DNA engineering and cloning method by uracil excision.. Nucleic Acids Res.

[11] Li MZ, Elledge SJ (2005). MAGIC, an in vivo genetic method for the rapid construction of recombinant DNA molecules.. Nat Genet.

[12] Li MZ, Elledge SJ (2007). Harnessing homologous recombination in vitro to generate recombinant DNA via SLIC.. Nat Methods.

[13] Marsischky G, LaBaer J (2004). Many paths to many clones: a comparative look at high-throughput cloning methods.. Genome Res.

[14] Klock HE, Koesema EJ, Knuth MW, Lesley SA (2008). Combining the polymerase incomplete primer extension method for cloning and mutagenesis with microscreening to accelerate structural genomics efforts.. Proteins.

[15] Nisson PE, Rashtchian A, Watkins PC (1991). Rapid and efficient cloning of Alu-PCR products using uracil DNA glycosylase.. PCR Methods Appl.

[16] Prodromou C, Pearl LH (1992). Recursive PCR: a novel technique for total gene synthesis.. Protein Eng.

[17] Stemmer WP, Crameri A, Ha KD, Brennan TM, Heyneker HL (1995). Single-step assembly of a gene and entire plasmid from large numbers of oligodeoxyribonucleotides.. Gene.

[18] Tian J, Gong H, Sheng N, Zhou X, Gulari E (2004). Accurate multiplex gene synthesis from programmable DNA microchips.. Nature.

[19] Smith HO, Hutchison CA, Pfannkoch C, Venter JC (2003). Generating a synthetic genome by whole genome assembly: phiX174 bacteriophage from synthetic oligonucleotides.. Proc Natl Acad Sci U S A.

[20] Kranz RG, Gabbert KK, Madigan MT (1997). Positive selection systems for discovery of novel polyester biosynthesis genes based on fatty acid detoxification.. Appl Environ Microbiol.

[21] Kung SS, Chuang YC, Chen CH, Chien CC (2007). Isolation of polyhydroxyalkanoates-producing bacteria using a combination of phenotypic and genotypic approach.. Lett Appl Microbiol.

