# Supplementary material for: Circular Polymerase Extension Cloning of Complex Gene Libraries and Pathways
Source: PLoS One. 2009 Jul 30;4(7):e6441. doi: 10.1371/journal.pone.0006441 (PMC2713398; doi:10.1371/journal.pone.0006441)
Supplement: Methods S1 — (0.09 MB DOC) [file pone.0006441.s001.doc]

**Circular Polymerase Extension Cloning of Complex Gene Libraries and Pathways**

**Jiayuan Quan & Jingdong Tian**

**Supplementary Methods S1**

**Bacterial strains, Media, Chemicals and Enzymes**

*E. coli* GC5 or DH5α competent cells were used for all CPEC clonings. LB (Miller) agar plates (Sigma) with appropriate antibiotics were used for culturing bacteria after transformation. Antibiotics were used at the following concentrations: carbenicillin (Cellgro) 100 µg/ml, kanamycin (Sigma) 30 µg/ml, and chloramphenicol (Sigma) 20 µg/ml. Phusion High-Fidelity DNA polymerase (Finnzymes) was used for CPEC reactions and Taq polymerase for single-colony PCR. The E.Z.N.A gel extraction kit (Omega Bio-Tek) was used for DNA purification.

**Preparation of Vectors and Inserts**

The pUC19stop vector was constructed by first inserting a stop codon TAA after nucleotide position 425 of pUC19 plasmid (Invitrogen) and then moving the multiple cloning site (MCS) out of the open reading frame of the *lacZα* gene by copying the part of the *lacZα* gene before MCS (from 455 bp to 469 bp) to its behind (after 394 bp) in order to join the rest of the *lacZα* gene into one intact piece. The pUC19stop plasmid was linearized and amplified by PCR using primers SOSH6-L and SOSH6-R. pAcGFP1N1 vector was linearized and amplified by PCR using primers pAcGFP1N1Fw3 and pAcGFP1N1Rv3. pASK was amplified from pASK-IBA7C vector (Cayman Chemical) by PCR using primers pASKFw2 and pASKRv. PhaAB was amplified from a previously constructed plasmid phaCAB Topo 15 by PCR using primers phaABFw and phaABRv. The terminator was amplified from commercial vector by PCR using primers TermFw and TermRv. Cat2phaC was amplified from a previously constructed plasmid pSOS-cat2phaC by PCR using primers cat2phaCFw and cat2phaCRv2.

The inserts of the *lacZα* and the HIV *gp120* gene libraries were obtained from other projects in the lab, which were made by substituting original codons with selected synonymous codons used in *E. coli.*. Primers LacZH-L and LacZH-R were used for amplifying the *lacZα* library. The HIV gp140 gene was split into two fragments, VacF1 and VacF2. Primer pairs GP140-R/GP140-28L and GP140L/GP140-29R were used to amplify VacF1 and VacF2, respectively. GP140-28L and GP140-29R were complementary with each other.

**List of** PCR templates and primers

| PCR products | Templates | Primers |
| --- | --- | --- |
| 2386 bp pUC19stop PCR product | pUC19stop | SOSH6-L, SOSH6-R |
| 4746 bp pAcGFP1N1 PCR product | pAcGFP1N1 | pAcGFP1N1Fw3, pAcGFP1N1Rv3 |
| 2959 bp pASK PCR product | pASK-IBA7C | pASKFw2, pASKRv |
| 2040 bp phaAB | phaCAB Topo 15 | phaABFw, phaABRv |
| 171bp terminator | J04450 | TermFw, TermRv |
| 3280 bp Cat2phaC | pSOS-cat2phaC | cat2phaCFw, cat2phaCRv2 |
| 307 bp LacZhis6 | Assembled LacZhis6 library | LacZH-L, LacZH-R |
| 889 bp VacF1 | Assembled VacF1 library | GP140-R, GP140-28L |
| 882 bp VacF2 | Assembled VacF2 library | GP140-L, GP140-29R |
| 592 bp LacZhis6 single-colony PCR product | pUC19stop-LacZhis6 | pUC19seqFw, pUC19seqRv |
| 2029 bp gp140 single-colony PCR product | pAcGFP1N1-gp140 | pAcGFP1N1seqFw, GFPRv |

**List of PCR primer sequences (also the overlapping regions between insert and vector fragments)**

| Primer name | Primer sequences |
| --- | --- |
| SOSH6-L | CAA TTT CAC ACA GGA AAC AGC TAT G |
| SOSH6-R | TAA CTA GTG GTG GTG ATG ATG ATG TGC |
| pAcGFP1N1Fw3 | GCT GTG GTA TGT TAA CTA TCG TAC GCG GGA TCC ACC GGT CTT G |
| pAcGFP1N1Rv3 | GGG CCC ACC GAA CGC CAT GAA TTC GAA GCT TGA GCT |
| pASKFw2 | CAA AGC cAA ggc atg aCC CTC GAG GTC GAC CTG CAG |
| pASKRv | CTT TCA ATG GTT GCC CTC GTT ATC TAG ATT TTT GTC G |
| phaABFw | AGA TAA CGA GGG CAA CCA TTG AAA GGA CTA CAC AAT GAC TGA CG |
| phaABRv | GCT CTA GTA TCA GCC CAT ATG CAG GCC GCC |
| TermFw | CTG CAT ATG GGC TGA TAC TAG AGC CAG GCA TCA AAT AAA ACG |
| TermRv | GCT CAC TGC CCG CTT TCC ATA TAA ACG CAG AAA G |
| cat2phaCFw | TGG AAA GCG GGC AGT GAG CGC |
| cat2phaCRv2 | CGA GGG TCA TGC CTT GGC TTT GAC GTA TCG C |
| LacZH-L | CAA TTT CAC ACA GGA AAC AGC TAT G |
| LacZH-R | TAA CTA GTG GTG GTG ATG ATG ATG TGC |
| GP140-R | CGT ACG CTA GTT AAC CTA CCA SAG C |
| GP140-28L | GAC ATC ATC GGC GAC ATC C |
| GP140-L | GCG TTC GGT GGG CCC AAC |
| GP140-29R | GGA TGT CGC CGA TGA TG |
| pUC19seqFw | GCA GCT GGC ACG ACA GGT TTC |
| pUC19seqRv | CGT CAT CAC CGA AAC GCG CGA |
| pAcGFP1N1seqFw | CAT TGA CGC AAA TGG GCG GTA GG |
| GFPRv | TTG CCG GTG GTG CAG ATG AAC |

**List of construct sequences:**

Note: Bold capital letters in DNA sequences indicate overlapping regions between fragments. The sequences in codon libraries are collectively represented using degenerate codons in capital letters.

**>pSOS-LacZhis6 plasmid (circular)**

gacgaaagggcctcgtgatacgcctatttttataggttaatgtcatgataataatggtttcttagacgtcaggtggcacttttcggggaaatgtgcgcggaacccctatttgtttatttttctaaatacattcaaatatgtatccgctcatgagacaataaccctgataaatgcttcaataatattgaaaaaggaagagtatgagtattcaacatttccgtgtcgcccttattcccttttttgcggcattttgccttcctgtttttgctcacccagaaacgctggtgaaagtaaaagatgctgaagatcagttgggtgcacgagtgggttacatcgaactggatctcaacagcggtaagatccttgagagttttcgccccgaagaacgttttccaatgatgagcacttttaaagttctgctatgtggcgcggtattatcccgtattgacgccgggcaagagcaactcggtcgccgcatacactattctcagaatgacttggttgagtactcaccagtcacagaaaagcatcttacggatggcatgacagtaagagaattatgcagtgctgccataaccatgagtgataacactgcggccaacttacttctgacaacgatcggaggaccgaaggagctaaccgcttttttgcacaacatgggggatcatgtaactcgccttgatcgttgggaaccggagctgaatgaagccataccaaacgacgagcgtgacaccacgatgcctgtagcaatggcaacaacgttgcgcaaactattaactggcgaactacttactctagcttcccggcaacaattaatagactggatggaggcggataaagttgcaggaccacttctgcgctcggcccttccggctggctggtttattgctgataaatctggagccggtgagcgtgggtctcgcggtatcattgcagcactggggccagatggtaagccctcccgtatcgtagttatctacacgacggggagtcaggcaactatggatgaacgaaatagacagatcgctgagataggtgcctcactgattaagcattggtaactgtcagaccaagtttactcatatatactttagattgatttaaaacttcatttttaatttaaaaggatctaggtgaagatcctttttgataatctcatgaccaaaatcccttaacgtgagttttcgttccactgagcgtcagaccccgtagaaaagatcaaaggatcttcttgagatcctttttttctgcgcgtaatctgctgcttgcaaacaaaaaaaccaccgctaccagcggtggtttgtttgccggatcaagagctaccaactctttttccgaaggtaactggcttcagcagagcgcagataccaaatactgtccttctagtgtagccgtagttaggccaccacttcaagaactctgtagcaccgcctacatacctcgctctgctaatcctgttaccagtggctgctgccagtggcgataagtcgtgtcttaccgggttggactcaagacgatagttaccggataaggcgcagcggtcgggctgaacggggggttcgtgcacacagcccagcttggagcgaacgacctacaccgaactgagatacctacagcgtgagctatgagaaagcgccacgcttcccgaagggagaaaggcggacaggtatccggtaagcggcagggtcggaacaggagagcgcacgagggagcttccagggggaaacgcctggtatctttatagtcctgtcgggtttcgccacctctgacttgagcgtcgatttttgtgatgctcgtcaggggggcggagcctatggaaaaacgccagcaacgcggcctttttacggttcctggccttttgctggccttttgctcacatgttctttcctgcgttatcccctgattctgtggataaccgtattaccgcctttgagtgagctgataccgctcgccgcagccgaacgaccgagcgcagcgagtcagtgagcgaggaagcggaagagcgcccaatacgcaaaccgcctctccccgcgcgttggccgattcattaatgcagctggcacgacaggtttcccgactggaaagcgggcagtgagcgcaacgcaattaatgtgagttagctcactcattaggcaccccaggctttacactttatgcttccggctcgtatgttgtgtggaattgtgagcggataa**CAATTTCACACAGGAAACAGCTATG**accatgattacgctggccgtcgttttacaacgtcgtgactgggaaaaccctggcgttacccaacttaatcgccttgcagcacatccccctttcgccagctggcgtaatagcgaagaggcccgcaccgatcgcccttcccaacagttgcgcagcctgaatggcgaatggcgcctgatgcggtattttctccttacgcatctgtgcggtatttcacaccgcatatggtgcactctcagtacaatctgctctgatgccgca**CATCATCATCACCACCACTAGTTA**agccagccccgacacccgccaacacccgctgacgcgccctgacgggcttgtctgctcccggcatccgcttacagacaagctgtgaccgtctccgggagctgcatgtgtcagaggttttcaccgtcatcaccgaaacgcgcga

**>pAcGFP1N1MATG-Vac plasmid (circular)**

tagttattaatagtaatcaattacggggtcattagttcatagcccatatatggagttccgcgttacataacttacggtaaatggcccgcctggctgaccgcccaacgacccccgcccattgacgtcaataatgacgtatgttcccatagtaacgccaatagggactttccattgacgtcaatgggtggagtatttacggtaaactgcccacttggcagtacatcaagtgtatcatatgccaagtacgccccctattgacgtcaatgacggtaaatggcccgcctggcattatgcccagtacatgaccttatgggactttcctacttggcagtacatctacgtattagtcatcgctattaccatggtgatgcggttttggcagtacatcaatgggcgtggatagcggtttgactcacggggatttccaagtctccaccccattgacgtcaatgggagtttgttttggcaccaaaatcaacgggactttccaaaatgtcgtaacaactccgccccattgacgcaaatgggcggtaggcgtgtacggtgggaggtctatataagcagagctggtttagtgaaccgtcagatccgctagcgctaccggactcagatctcgagctcaagcttcgaattcatg**GCGTTCGGTGGGCCC**AACCTSTGGGTSACSGTSTACTACGGYGTSCCGGTSTGGAAGGARGCRAACACSACSCTSTTCTGCGCRTCSGAYGCRAAGGCRTACGAYACSGARGTSCACAACGTSTGGGCRACSCACGCRTGCGTSCCGACSGAYCCGAACCCGCAGGARATCGTSCTSGARAACGTSACSGARAACTTCAACATGTGGAAGAACAACATGGTSGARCAGATGCACGARGAYATCATCTCSCTSTGGGAYCAGTCSCTSAAGCCGTGCGTSAAGCTSACSCCGCTSTGCGTSACSCTSAACTGCACSAACGTSAACGTSACSAACACSACSAACAACACSGARGARAAGGGYGARATCAAGAACTGCTCSTTCAACATCACSACSGARATCCGYGAYAAGAAGCAGAAGGTSTACGCRCTSTTCTACCGYCTSGAYGTSGTSCCGATCGAYGAYAACAACAACAACTCSTCSAACTACCGYCTSATCAACTGCAACACSTCSGCRATCACSCAGGCRTGCCCGAAGGTSTCSTTCGARCCGATCCCGATCCACTACTGCGCRCCGGCRGGYTTCGCRATCCTSAAGTGCAACGAYAAGAAGTTCAACGGYACSGGYCCGTGCAAGAACGTSTCSACSGTSCAGTGCACSCACGGYATCAAGCCGGTSGTSTCSACSCAGCTSCTSCTSAACGGYTCSCTSGCRGARGARGARATCATCATCCGYTCSGARAACATCACSAACAACGCRAAGACSATCATCGTSCAGCTSAACGARTCSGTSGARATCAACTGCACSCGYCCGAACAACAACACSCGYAAGTCSATCCGYATCGGYCCGGGYCAGGCRTTCTACGCRACSGGYGAYATcatcggcgacatccgccagGCRCACTGCAACATCTCSGGYACSAAGTGGAACAAGACSCTSCAGCAGGTSGCRAAGAAGCTSCGYGARCACTTCAACAACAAGACSATCATCTTCAAGCCGTCSTCSGGYGGYGAYCTSGARATCACSACSCACTCSTTCAACTGCCGYGGYGARTTCTTCTACTGCCGYAAGTCSATCCGYATCCAGCGYGGYCCGGGYCGYGCRTTCGTSACSATCGGYAAGATCATCGGYTGCCGYATCAAGCAGATCATCAACATGTGGCAGGGYGTSGGYCAGGCRATGTACGCRCCGCCGATCGARGGYAAGATCACSTGCAAGTCSAACATCACSGGYCTSCTSCTSACSCGYGAYGGYGGYAACAACAACACSAACGARACSGARATCTTCCGYCCGGGYGGYGGYGAYATGCGYGAYAACTGGCGYTCSGARCTSTACAAGTACAAGGTSGTSAAGATCGARCCGCTSGGYGTSGCRCCGACSAAGGCRAAGCTSACSGTSCAGGCRCGYCAGCTSCTSTCSGGYATCGTSCAGCAGCAGTCSAACCTSCTSCGYGCRATCGARGCRCAGCAGCACCTSCTSCAGCTSACSGTSTGGGGYATCAAGCAGCTSCAGGCRCGYGTSCTSGCRGTSGARCGYTACCTSAAGGAYCAGCAGCTSGARATCTGGGAYAACATGACSTGGATGGARTGGGARCGYGARATCAACAACTACACSGAYATCATCTACTCSCTSATCGARGARTCSCAGAACCAGCAGGARAAGAACGARCAGGARCTSCTSGCRCTSGAYAAGTGGGCRTCSCTSTGGAACTGGTTCGAYATCACSAACTG**GCTGTGGTATGTTAACTATCGTACG**cgggatccaccggtcttggtgagcaagggcgccgagctgttcaccggcatcgtgcccatcctgatcgagctgaatggcgatgtgaatggccacaagttcagcgtgagcggcgagggcgagggcgatgccacctacggcaagctgaccctgaagttcatctgcaccaccggcaagctgcctgtgccctggcccaccctggtgaccaccctgagctacggcgtgcagtgcttctcacgctaccccgatcacatgaagcagcacgacttcttcaagagcgccatgcctgagggctacatccaggagcgcaccatcttcttcgaggatgacggcaactacaagtcgcgcgccgaggtgaagttcgagggcgataccctggtgaatcgcatcgagctgaccggcaccgatttcaaggaggatggcaacatcctgggcaataagatggagtacaactacaacgcccacaatgtgtacatcatgaccgacaaggccaagaatggcatcaaggtgaacttcaagatccgccacaacatcgaggatggcagcgtgcagctggccgaccactaccagcagaatacccccatcggcgatggccctgtgctgctgcccgataaccactacctgtccacccagagcgccctgtccaaggaccccaacgagaagcgcgatcacatgatctacttcggcttcgtgaccgccgccgccatcacccacggcatggatgagctgtacaagtgagcggccgcgactctagatcataatcagccataccacatttgtagaggttttacttgctttaaaaaacctcccacacctccccctgaacctgaaacataaaatgaatgcaattgttgttgttaacttgtttattgcagcttataatggttacaaataaagcaatagcatcacaaatttcacaaataaagcatttttttcactgcattctagttgtggtttgtccaaactcatcaatgtatcttaaggcgtaaattgtaagcgttaatattttgttaaaattcgcgttaaatttttgttaaatcagctcattttttaaccaataggccgaaatcggcaaaatcccttataaatcaaaagaatagaccgagatagggttgagtgttgttccagtttggaacaagagtccactattaaagaacgtggactccaacgtcaaagggcgaaaaaccgtctatcagggcgatggcccactacgtgaaccatcaccctaatcaagttttttggggtcgaggtgccgtaaagcactaaatcggaaccctaaagggagcccccgatttagagcttgacggggaaagccggcgaacgtggcgagaaaggaagggaagaaagcgaaaggagcgggcgctagggcgctggcaagtgtagcggtcacgctgcgcgtaaccaccacacccgccgcgcttaatgcgccgctacagggcgcgtcaggtggcacttttcggggaaatgtgcgcggaacccctatttgtttatttttctaaatacattcaaatatgtatccgctcatgagacaataaccctgataaatgcttcaataatattgaaaaaggaagagtcctgaggcggaaagaaccagctgtggaatgtgtgtcagttagggtgtggaaagtccccaggctccccagcaggcagaagtatgcaaagcatgcatctcaattagtcagcaaccaggtgtggaaagtccccaggctccccagcaggcagaagtatgcaaagcatgcatctcaattagtcagcaaccatagtcccgcccctaactccgcccatcccgcccctaactccgcccagttccgcccattctccgccccatggctgactaattttttttatttatgcagaggccgaggccgcctcggcctctgagctattccagaagtagtgaggaggcttttttggaggcctaggcttttgcaaagatcgatcaagagacaggatgaggatcgtttcgcatgattgaacaagatggattgcacgcaggttctccggccgcttgggtggagaggctattcggctatgactgggcacaacagacaatcggctgctctgatgccgccgtgttccggctgtcagcgcaggggcgcccggttctttttgtcaagaccgacctgtccggtgccctgaatgaactgcaagacgaggcagcgcggctatcgtggctggccacgacgggcgttccttgcgcagctgtgctcgacgttgtcactgaagcgggaagggactggctgctattgggcgaagtgccggggcaggatctcctgtcatctcaccttgctcctgccgagaaagtatccatcatggctgatgcaatgcggcggctgcatacgcttgatccggctacctgcccattcgaccaccaagcgaaacatcgcatcgagcgagcacgtactcggatggaagccggtcttgtcgatcaggatgatctggacgaagagcatcaggggctcgcgccagccgaactgttcgccaggctcaaggcgagcatgcccgacggcgaggatctcgtcgtgacccatggcgatgcctgcttgccgaatatcatggtggaaaatggccgcttttctggattcatcgactgtggccggctgggtgtggcggaccgctatcaggacatagcgttggctacccgtgatattgctgaagagcttggcggcgaatgggctgaccgcttcctcgtgctttacggtatcgccgctcccgattcgcagcgcatcgccttctatcgccttcttgacgagttcttctgagcgggactctggggttcgaaatgaccgaccaagcgacgcccaacctgccatcacgagatttcgattccaccgccgccttctatgaaaggttgggcttcggaatcgttttccgggacgccggctggatgatcctccagcgcggggatctcatgctggagttcttcgcccaccctagggggaggctaactgaaacacggaaggagacaataccggaaggaacccgcgctatgacggcaataaaaagacagaataaaacgcacggtgttgggtcgtttgttcataaacgcggggttcggtcccagggctggcactctgtcgataccccaccgagaccccattggggccaatacgcccgcgtttcttccttttccccaccccaccccccaagttcgggtgaaggcccagggctcgcagccaacgtcggggcggcaggccctgccatagcctcaggttactcatatatactttagattgatttaaaacttcatttttaatttaaaaggatctaggtgaagatcctttttgataatctcatgaccaaaatcccttaacgtgagttttcgttccactgagcgtcagaccccgtagaaaagatcaaaggatcttcttgagatcctttttttctgcgcgtaatctgctgcttgcaaacaaaaaaaccaccgctaccagcggtggtttgtttgccggatcaagagctaccaactctttttccgaaggtaactggcttcagcagagcgcagataccaaatactgtccttctagtgtagccgtagttaggccaccacttcaagaactctgtagcaccgcctacatacctcgctctgctaatcctgttaccagtggctgctgccagtggcgataagtcgtgtcttaccgggttggactcaagacgatagttaccggataaggcgcagcggtcgggctgaacggggggttcgtgcacacagcccagcttggagcgaacgacctacaccgaactgagatacctacagcgtgagctatgagaaagcgccacgcttcccgaagggagaaaggcggacaggtatccggtaagcggcagggtcggaacaggagagcgcacgagggagcttccagggggaaacgcctggtatctttatagtcctgtcgggtttcgccacctctgacttgagcgtcgatttttgtgatgctcgtcaggggggcggagcctatggaaaaacgccagcaacgcggcctttttacggttcctggccttttgctggccttttgctcacatgttctttcctgcgttatcccctgattctgtggataaccgtattaccgccatgcat

**>Bioplastic pathway plasmid (circular)**

aggtcgacctgcagggggaccatggtctctgatatctaactaagcttgacctgtgaagtgaaaaatggcgcacattgtgcgacattttttttgtctgccgtttaccgctactgcgtcacggatctccacgcgccctgtagcggcgcattaagcgcggcgggtgtggtggttacgcgcagcgtgaccgctacacttgccagcgccctagcgcccgctcctttcgctttcttcccttcctttctcgccacgttcgccggctttccccgtcaagctctaaatcgggggctccctttagggttccgatttagtgctttacggcacctcgaccccaaaaaacttgattagggtgatggttcacgtagtgggccatcgccctgatagacggtttttcgccctttgacgttggagtccacgttctttaatagtggactcttgttccaaactggaacaacactcaaccctatctcggtctattcttttgatttataagggattttgccgatttcggcctattggttaaaaaatgagctgatttaacaaaaatttaacgcgaattttaacaaaatttggcgaaaatgagacgttgatcggcacgtaagaggttccaactttcaccataatgaaataagatcactaccgggcgtattttttgagttatcgagattttcaggagctaaggaagctaaaatggagaaaaaaatcactggatataccaccgttgatatatcccaatggcatcgtaaagaacattttgaggcatttcagtcagttgctcaatgtacctataaccagaccgttcagctggatattacggcctttttaaagaccgtaaagaaaaataagcacaagttttatccggcctttattcacattcttgcccgcctgatgaatgctcatccggagttccgtatggcaatgaaagacggtgagctggtgatatgggatagtgttcacccttgttacaccgttttccatgagcaaactgaaacgttttcatcgctctggagtgaataccacgacgatttccggcagtttctacacatatattcgcaagatgtggcgtgttacggtgaaaacctggcctatttccctaaagggtttattgagaatatgtttttcgtctcagccaatccctgggtgagtttcaccagttttgatttaaacgtggccaatatggacaacttcttcgcccccgttttcactatgggcaaatattatacgcaaggcgacaaggtgctgatgccgctggcgattcaggttcatcatgccgtttgtgatggcttccatgtcggcagaatgcttaatgaattacaacagtactgcgatgagtggcagggcggggcgtaataggaattaatgatgtctcgtttagataaaagtaaagtgattaacagcgcattagagctgcttaatgaggtcggaatcgaaggtttaacaacccgtaaactcgcccagaagctaggtgtagagcagcctacattgtattggcatgtaaaaaataagcgggctttgctcgacgccttagccattgagatgttagataggcaccatactcacttttgccctttagaaggggaaagctggcaagattttttacgtaataacgctaaaagttttagatgtgctttactaagtcatcgcgatggagcaaaagtacatttaggtacacggcctacagaaaaacagtatgaaactctcgaaaatcaattagcctttttatgccaacaaggtttttcactagagaatgcattatatgcactcagcgcagtggggcattttactttaggttgcgtattggaagatcaagagcatcaagtcgctaaagaagaaagggaaacacctactactgatagtatgccgccattattacgacaagctatcgaattatttgatcaccaaggtgcagagccagccttcttattcggccttgaattgatcatatgcggattagaaaaacaacttaaatgtgaaagtgggtcttaaaagcagcataacctttttccgtgatggtaacttcactagtttaaaaggatctaggtgaagatcctttttgataatctcatgaccaaaatcccttaacgtgagttttcgttccactgagcgtcagaccccgtagaaaagatcaaaggatcttcttgagatcctttttttctgcgcgtaatctgctgcttgcaaacaaaaaaaccaccgctaccagcggtggtttgtttgccggatcaagagctaccaactctttttccgaaggtaactggcttcagcagagcgcagataccaaatactgtccttctagtgtagccgtagttaggccaccacttcaagaactctgtagcaccgcctacatacctcgctctgctaatcctgttaccagtggctgctgccagtggcgataagtcgtgtcttaccgggttggactcaagacgatagttaccggataaggcgcagcggtcgggctgaacggggggttcgtgcacacagcccagcttggagcgaacgacctacaccgaactgagatacctacagcgtgagctatgagaaagcgccacgcttcccgaagggagaaaggcggacaggtatccggtaagcggcagggtcggaacaggagagcgcacgagggagcttccagggggaaacgcctggtatctttatagtcctgtcgggtttcgccacctctgacttgagcgtcgatttttgtgatgctcgtcaggggggcggagcctatggaaaaacgccagcaacgcggcctttttacggttcctggccttttgctggccttttgctcacatgacccgacaCcatcgaatggccagatgattaattcctaatttttgttgacactctatcattgatagagttattttaccactccctatcagtgatagagaaaagtgaaatgaatagttcgacaaaaatct**agataacgagggcaaccattgaaag**gactacacaatgactgacgttgtcatcgtatccgccgcccgcaccgcggtcggcaagtttggcggctcgctggccaagatcccggcaccggaactgggtgccgtggtcatcaaggccgcgctggagcgcgccggcgtcaagccggagcaggtgagcgaagtcatcatgggccaggtgctgaccgccggttcgggccagaaccccgcacgccaggccgcgatcaaggccggcctgccggcgatggtgccggccatgaccatcaacaaggtgtgcggctcgggcctgaaggccgtgatgctggccgccaacgcgatcatggcgggcgacgccgagatcgtggtggccggcggccaggaaaacatgagcgccgccccgcacgtgctgccgggctcgcgcgatggtttccgcatgggcgatgccaagctggtcgacaccatgatcgtcgacggcctgtgggacgtgtacaaccagtaccacatgggcatcaccgccgagaacgtggccaaggaatacggcatcacacgcgaggcgcaggatgagttcgccgtcggctcgcagaacaaggccgaagccgcgcagaaggccggcaagtttgacgaagagatcgtcccggtgctgatcccgcagcgcaagggcgacccggtggccttcaagaccgacgagttcgtgcgccagggcgccacgctggacagcatgtccggcctcaagcccgccttcgacaaggccggcacggtgaccgcggccaacgcctcgggcctgaacgacggcgccgccgcggtggtggtgatgtcggcggccaaggccaaggaactgggcctgaccccgctggccacgatcaagagctatgccaacgccggtgtcgatcccaaggtgatgggcatgggcccggtgccggcctccaagcgcgccctgtcgcgcgccgagtggaccccgcaagacctggacctgatggagatcaacgaggcctttgccgcgcaggcgctggcggtgcaccagcagatgggctgggacacctccaaggtcaatgtgaacggcggcgccatcgccatcggccacccgatcggcgcgtcgggctgccgtatcctggtgacgctgctgcacgagatgaagcgccgtgacgcgaagaagggcctggcctcgctgtgcatcggcggcggcatgggcgtggcgctggcagtcgagcgcaaataaggaaggggttttccggggccgcgcgcggttggcgcggacccggcgacgataacgaagccaatcaaggagtggacatgactcagcgcattgcgtatgtgaccggcggcatgggtggtatcggaaccgccatttgccagcggctggccaaggatggctttcgtgtggtggccggttgcggccccaactcgccgcgccgcgaaaagtggctggagcagcagaaggccctgggcttcgatttcattgcctcggaaggcaatgtggctgactgggactcgaccaagaccgcattcgacaaggtcaagtccgaggtcggcgaggttgatgtgctgatcaacaacgccggtatcacccgcgacgtggtgttccgcaagatgacccgcgccgactgggatgcggtgatcgacaccaacctgacctcgctgttcaacgtcaccaagcaggtgatcgacggcatggccgaccgtggctggggccgcatcgtcaacatctcgtcggtgaacgggcagaagggccagttcggccagaccaactactccaccgccaaggccggcctgcatggcttcaccatggcactggcgcaggaagtggcgaccaagggcgtgaccgtcaacacggtctctccgggctatatcgccaccgacatggtcaaggcgatccgccaggacgtgctcgacaagatcgtcgcgacgatcccggtcaagcgcctgggcctgccggaagagatcgcctcgatctgcgcctggttgtcgtcggaggagtccggtttctcgaccggcgccgacttctcgctcaacggcggc**ctgcatatgggctgatactagagc**caggcatcaaataaaacgaaaggctcagtcgaaagactgggcctttcgttttatctgttgtttgtcggtgaacgctctctactagagtcacactggctcaccttcgggtgggcctttctgcgtttata**tggaaagcgggcagtgagc**gcaacgcaattaatgtgagttagctcactcattaggcaccccaggctttacactttatgcttccggctcgtatgttgtgtggaattgtgagcggataacaatttcacacaggaaacagctatgaccatgattacgccaagcttgcatgcctgcaggtcgactctaatggagtgggaagagatatataaagagaaactggtaactgcagaaaaagctgtttcaaaaatagaaaaccatagcagggtagtttttgcacatgcagtaggagaacccgtagatttagtaaatgcactagttaaaaataaggataattatataggactagaaatagttcacatggtagctatgggcaaaggtgaatatacaaaagagggtatgcaaagacattttagacataatgctttatttgtaggcggatgtactagagatgcagtaaattcaggaagagcagattatacaccttgttttttctatgaagtgccaagtttgtttaaagaaaaacgtttgcctgtagatgtagcacttattcaggtaagtgagccagataaatatggctactgcagttttggagtttccaatgactataccaagccagcagcagaaagtgctaagcttgtaattgcagaagtgaataaaaacatgccaagaactcttggagattcttttatacatgtatcagatattgattatatagtggaagcttcacacccattgttagaattgcagcctcctaaattgggagatgtagaaaaagccataggagaaaactgtgcatctttaattgaagatggagctactcttcagcttggaataggtgctataccagatgcggtacttttattcttaaagaacaaaaagaatttaggaatacattctgagatgatatcagatggtgtgatggaactggtgaaggcaggggttatcaataacaagaaaaagaccctccatccaggcaaaatagttgtaacatttttaatgggaacaaaaaaattatatgattttgtaaacaataatccaatggtagaaacttattctgtagattatgtaaataatccactggtaattatgaaaaatgacaatatggtttcaataaattcttgtgttcaagtagacttaatgggacaagtatgttctgaaagtataggattgaaacagataagtggagtgggaggccaggtagattttattagaggagctaatctatcaaagggtggaaaggctattatagctataccttccacagctggaaaaggaaaagtttcaagaataactccacttctagatactggtgctgcagttacaacttctagaaatgaagtagattatgtagttactgaatatggtgttgctcatcttaagggcaaaactttaagaaatagggcaagagctctaataaatatcgctcatccaaaattcagagaatcattaatgaatgaatttaaaaagagattgaggatcctttgacggcagagagacaatcaaatcatggcgaccggcaaaggcgcggcagcttccacgcaggaaggcaagtcccaaccattcaaggtcacgccggggccattcgatccagccacatggctggaatggtcccgccagtggcagggcactgaaggcaacggccacgcggccgcgtccggcattccgggcctggatgcgctggcaggcgtcaagatcgcgccggcgcagctgggtgatatccagcagcgctacatgaaggacttctcagcgctgtggcaggccatggccgagggcaaggccgaggccaccggtccgctgcacgaccggcgcttcgccggcgacgcatggcgcaccaacctcccatatcgcttcgctgccgcgttctacctgctcaatgcgcgcgccttgaccgagctggccgatgccgtcgaggccgatgccaagacccgccagcgcatccgcttcgcgatctcgcaatgggtcgatgcgatgtcgcccgccaacttccttgccaccaatcccgaggcgcagcgcctgctgatcgagtcgggcggcgaatcgctgcgtgccggcgtgcgcaacatgatggaagacctgacacgcggcaagatctcgcagaccgacgagagcgcgtttgaggtcggccgcaatgtcgcggtgaccgaaggcgccgtggtcttcgagaacgagtacttccagctgttgcagtacaagccgctgaccgacaaggtgcacgcgcgcccgctgctgatggtgccgccgtgcatcaacaagtactacatcctggacctgcagccggagagctcgctggtgcgccatgtggtggagcagggacatacggtgtttctggtgtcgtggcgcaatccggacgccagcatggccggcagcacctgggacgactacatcgagcacgcggccatccgcgccatcgaagtcgcgcgcgacatcagcggccaggacaagatcaacgtgctcggcttctgcgtgggcggcaccattgtctcgaccgcgctggcggtgctggccgcgcgcggcgagcacccggccgccagcgtcacgctgctgaccacgctgctggactttgccgacacgggcatcctcgacgtctttgtcgacgagggccatgtgcagttgcgcgaggccacgctgggcggcggcgccggcgcgccgtgcgcgctgctgcgcggccttgagctggccaataccttctcgttcttgcgcccgaacgacctggtgtggaactacgtggtcgacaactacctgaagggcaacacgccggtgccgttcgacctgctgttctggaacggcgacgccaccaacctgccggggccgtggtactgctggtacctgcgccacacctacctgcagaacgagctcaaggtaccgggcaagctgaccgtgtgcggcgtgccggtggacctggccagcatcgacgtgccgacctatatctacggctcgcgcgaagaccatatcgtgccgtggaccgcggcctatgcctcgaccgcgctgctggcgaacaagctgcgcttcgtgctgggtgcgtcgggccatatcgccggtgtgatcaacccgccggccaagaacaagcgcagccactggactaacgatgcgctgccggagtcgccgcagcaatggctggccggcgccatcgagcatcacggcagctggtggccggactggaccgcatggctggccgggcaggccggcgcgaaacgcgccgcgcccgccaactatggcaatgcgcgctatcgcgcaatcgaacccgcgcctgggcgatacgt**caaagccaaggcatgaccctcg**
